# A minimal region of the HSP90AB1 promoter is suitable for ubiquitous expression in different somatic tissues with applicability for gene therapy

**DOI:** 10.3389/fmolb.2023.1175407

**Published:** 2023-04-17

**Authors:** Michal Mielcarek, Mark Isalan

**Affiliations:** ^1^ Department of Life Sciences, Imperial College London, London, United Kingdom; ^2^ Imperial College Centre for Synthetic Biology, Imperial College London, London, United Kingdom

**Keywords:** Huntington’s disease, mouse models, gene therapy, promoter, aav, zinc finger therapeutics

## Abstract

Huntington’s disease (HD) is a multi-tissue failure disorder for which there is no cure. We have previously shown an effective therapeutic approach limited mainly to the central nervous system, based on a synthetic zinc finger (ZF) transcription repressor gene therapy, but it would be important to target other tissues as well. In this study, we identify a novel minimal HSP90AB1 promoter region that can efficiently control expression not only in the CNS but also in other affected HD tissues. This promoter-enhancer is effective in driving expression of ZF therapeutic molecules in both HD skeletal muscles and the heart, in the symptomatic R6/1 mouse model. Moreover, for the first time we show that ZF molecules repressing mutant HTT reverse transcriptional pathological remodelling in HD hearts. We conclude that this HSP90AB1 minimal promoter may be used to target multiple HD organs with therapeutic genes. The new promoter has the potential to be added to the portfolio of gene therapy promoters, for use where ubiquitous expression is needed.

## Introduction

**FIGURE 1:**
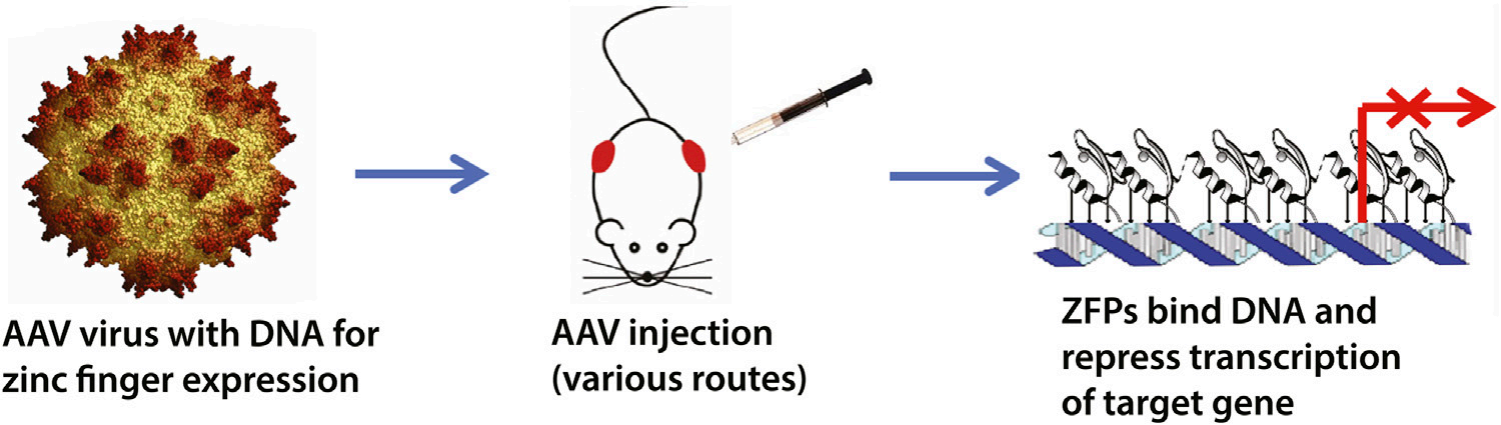
Schematic overview of the zinc finger transcription factor (ZF-TF) platform. We have previously designed zinc fingers to selectively target expanded CAG-repeats in Huntington’s disease (Garriga-Canut et al., 2012; Agustin-Pavon et al., 2016). The ZF-TFs are delivered as DNA in adeno-associated viruses (AAVs) and can be delivered by injection *via* various routes, including intrathecal and intravenous. The zinc fingers are expressed inside cells and bind their DNA targets to repress transcription *via* KRAB repressor domains fused to the zinc fingers.

The zinc finger transcription factor (ZF-TF) platform is a broadly-applicable technology to silence lethal mutations at their source-at the DNA level Figure 1. We have previously shown its application in Huntington’s disease (HD), where a ZF synthetic construct effectively and selectively repressed the expanded CAG sequence within the mutant *Huntingin* allele, which is the source of HD (Garriga-Canut et al., 2012; Agustin-Pavon et al., 2016). Unlike CRISPR nuclease approaches, synthetic TFs are inherently safer because they do not cut DNA, which leads to permanent effects (Papworth et al., 2003; Reynolds et al., 2003; Tan et al., 2003). Furthermore, non-replicating, non-integrating vectors such as recombinant adeno-associated viruses (rAAVs) have now improved efficiency and safety, as well as providing clinically practical delivery routes, including intravenous injection (Au et al., 2021).

Huntington’s disease is a fatal neurodegenerative disorder for which there is currently no effective therapy (Zielonka et al., 2015). It is a genetic disorder caused by an abnormal CAG expansion that is translated into a polyQ track within the Huntingtin protein, leading to a decline in movement, cognitive and psychiatric abilities, due to the central nervous system malfunction (Walker, 2007). HD has been recognised as a multi-system disorder (Mielcarek, 2015) due to mounting evidence of peripheral tissue pathologies and a high prevalence of non-psychiatric comorbidities in HD patients (Zielonka et al., 2020). These pathologies have been also widely described in a number HD mouse models with an apparent malfunction of skeletal and cardiac muscles (Critchley et al., 2018; Mielcarek and Isalan, 2021a). HD-related cardiomyopathy has been shown to be manifested by a pathological remodelling of foetal gene transcripts (Mielcarek et al., 2014a) and impaired metabolism of nucleotides at the molecular level (Toczek et al., 2016a; Toczek et al., 2016b). There is also a significant malfunction of HD skeletal muscles that has been shown to be characterised by a progressive impairment of the contractile characteristics of the hind limb muscles, accompanied by a significant loss of motor units (Mielcarek et al., 2015). Moreover, there is a significant deterioration in energy metabolism, along with a decreased oxidation activity (Zielonka et al., 2014a; Mielcarek et al., 2015), that has been linked to an altered purine metabolism transcriptome (Mielcarek et al., 2017). Hence, one may conclude that an effective therapy for HD patients should not be only restricted to the central nervous system.

Advances in synthetic gene regulation show promise in efficient delivery of artificial transcription factors (TFs) on recombinant AAV viruses, by direct injection. Our previous study showed that an endogenous promoter, based on a neuronal specific enolase (NSE) promoter-enhancer, was sufficient for stable long-term expression of a synthetic ZF targeting a mutant (but not wild type) Htt allele (Agustin-Pavon et al., 2016). However, because of the neuronal nature of the promoter, therapeutic expression was restricted to the central nervous system only. In the current study, we aimed to define a novel minimal promoter that would be sufficient to drive long-term expression of synthetic ZFs, up to 6 months, in the various cell types (tissues) that are mainly affected in HD, like skeletal muscles and heart. Importantly, this study was also driven by the idea of testing different delivery routes that are clinically practical (e.g., intrathecal, intramuscular, intravenous), allowing the realistic prospect of future translation into humans.

## Results

We have previously shown that synthetic ZF transcription factors efficiently silenced mutant HTT transcripts in either specific brain regions (Garriga-Canut et al., 2012; Agustin-Pavon et al., 2016), or in the whole brain (Agustin-Pavon et al., 2016), in various HD mouse models. In these earlier studies, we used two types of promoters to control the ZF expression. First, we used an exogenous CMV-enhanced CAG promoter that likely became methylated and inactivated after several weeks (Garriga-Canut et al., 2012). Second, we switched to an endogenous NSE (Neuronal Specific Enolase) promoter, which drove ZF expression in the CNS for at least 6 months (Agustin-Pavon et al., 2016). HD has been recognised as a multi-system disorder, affecting virtually all tissues (Mielcarek, 2015; Mielcarek and Isalan, 2021a) (due to ubiquitous expression of *HTT* transcripts (Li et al., 1993; Saudou and Humbert, 2016)). Therefore, here we aimed to characterise a new endogenous promoter with ubiquitous tissue expression, while also controlling expression of the therapeutic ZF in important tissues that are pathologically affected by mutant *HTT,* including the CNS, skeletal muscles and the heart.

To find a candidate ubiquitous endogenous promoter, we mined new endogenous promoters-enhancers in the literature. For instance, a recent RNA-seq study explored gene expression in the striatum and cortex, in WT and R6/2 mice, and found many genes consistently-upregulated in all four sample types (Vashishtha et al., 2013). The promoter-enhancer regions from some of these genes might potentially be good candidates to drive therapeutic gene expression. However, the functional promoter-enhancer regions remained to be characterised.

We started with a preliminary analysis *in silico* for alternative promoter candidates and found 8 that are in the top 20 most-expressed genes in all conditions in Vashishtha *et al.* (Vashishtha et al., 2013) (conditions: cortex and striatum, 8 and 12 week-old mice, R6/2 and WT; Supplementary Table S3 therein). In order of expression, these highly-expressed genes are: Tmsb4x (NCBI Gene ID: 19241), Snap25 (20614), Fth1 (14319), Cst3 (13010), Cpe (12876), Hsp90ab1 (15516), Calm1 (12313) and Rtn1 (104001).

Out of this list, we selected the ubiquitous gene promoter Hsp90ab1 (hereafter abbreviated as HSP90) because the gene product is reported as being strongly expressed in a large variety of cell types in various organisms (see NCBI Gene ID: 15516). This gene promoter belongs to heat shock protein HSP90: the Hsp90beta isoform is constitutively expressed, whereas the Hsp90alpha isoforms is expressed under stress. As the promoter/enhancer had not been characterised, we set out to test a potential region in the mouse promoter (NCBI 15516 NC_000083.6). This region shares homology with the counterpart human promoter. Because we were constrained by the 1810bp packaging limit of our AAV-ZF vector, we selected a region of 1.7k upstream of the TSS, plus 95bp of the transcript, while remaining under the 1810bp AAV packaging limit. Flanking NheI sites were added for cloning into AAV vector (See Supplementary Figure S1). We therefore selected this promoter for vectorisation in AAV for studies of expression in mice.

**FIGURE 2:**
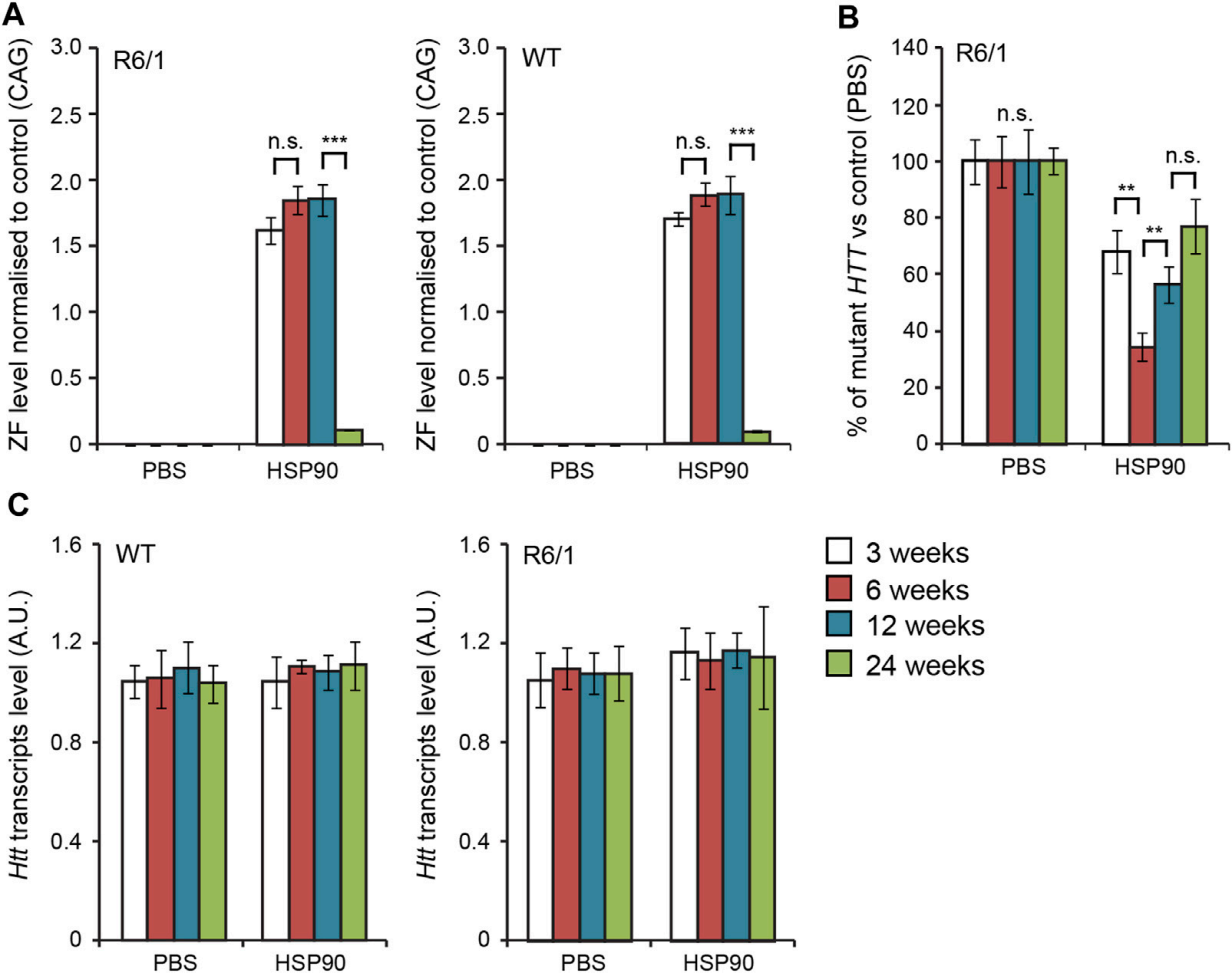
Long-term effects of bilateral intraventricular injection of AAV expressing mZF-KRAB zinc finger under the HSP90 promoter. **(A)** Zinc finger expression over time. mZF-KRAB transcript levels from whole brains were assayed by qRT-PCR at 3, 6, 12, and 24 weeks after viral (or PBS control) injections, in WT or R6/1 neonates. **(B)** Zinc finger repression of mutant Huntingtin in R6/1 mice. *mut HTT* (exon 1) expression levels in the whole brain samples from the various treatments were compared to transcript levels in PBS controls, by qRT-PCR. **(C)** Verification of lack of cross-reactivity of mZF-KRAB with short WT *HTT* alleles. WT *HTT* (exon 1) expression levels were quantified in the same treatment samples as above. All transcript levels were normalized to three housekeeping genes (see Supplementary Figure S2). Error bars are S.E.M (*n* = 4). **p < 0.01, ***p < 0.001, n.s. = not significant.

In the first set of experiments, we validated the expression of our previous anti-mutant Huntingtin zinc finger, mZF-KRAB (Agustin-Pavon et al., 2016), under the control of the HSP90 promoter, in the CNS of the R6/1 mouse model. We used bilateral intraventricular injections of AAV2/9 carrying mZF-KRAB under the HSP90 promoter, in neonatal pups of either wild type or R6/1 mice, as previously described (Agustin-Pavon et al., 2016). We monitored expression of mZF-KRAB transcripts at several time-points: 3, 6, 12 and 24 weeks post-single injection into the neonatal mice. We found consistent expression of the mZF-KRAB mRNA at 3, 6 and 12 weeks post single injection, followed by a drop at 24 weeks, both in the wild type and R6/1 cohort of mice Figure 2A. As expected, the mZF-KRAB significantly repressed mutant *Htt* transcript levels by approximately 20% at 3 weeks, 60% at 6 weeks, 40% at 12 weeks and 20% at 24 weeks post single injection Figure 2B. Importantly, the wild-type alleles of *Htt* remained unchanged at all time-points in both wild-type and R6/1 mice, indicating mutant allele selectivity Figure 2C. We normalised expression of mZF-KRAB, mutant *Htt* and wild-type *Htt* transcripts to a previously selected set of housekeeping genes (Agustin-Pavon et al., 2016), Supplementary Figure S2.

**FIGURE 3:**
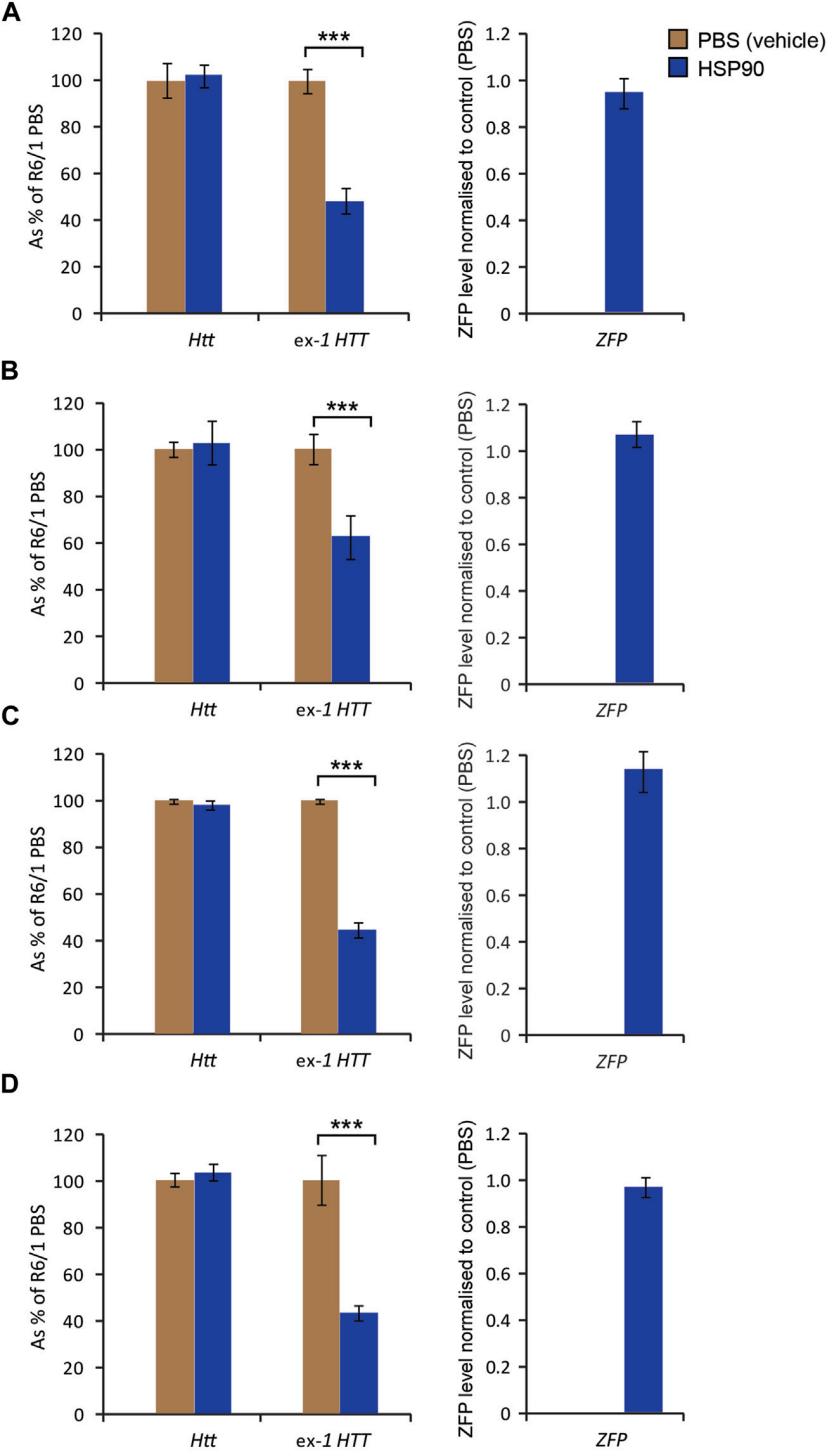
Effects of bilateral intraventricular injection of AAV expressing mZF-KRAB under the HSP90 promoter in specific brain regions 6 weeks post an injection. Unchanged expression WT HTT transcripts, as well the repression of mutant Huntingtin mut-Exon1, by targeted zinc finger expression. mZF-KRAB (ZFP) transcript levels are from dissected brain regions of the R6/1 mice, treated with either AAV2/9 (carrying mZF-KRAB under the HSP90 promoter) or PBS (control). The specific brain regions are: **(A)** cortex, **(B)** cerebellum, **(C)** striatum, **(D)** hippocampus. All transcript levels were normalized to three housekeeping genes see Supplementary Figure S3. Error bars are S.E.M (*n* = 4). ***p < 0.001.

Next, we examined the mZF-KRAB expression in the specific brain regions 6 weeks post a single bilateral intraventricular injection of AAV2/9-ZF into neonatal R6/1 mice. We found mZF-KRAB mRNA to be detectable in all examined brain regions: Figure 3A) cortex, Figure 3B) cerebellum, Figure 3C) striatum, Figure 3D) hippocampus. Consequently, we detected a significant reduction of mutant *Htt* transcripts but not wild-type *Htt* on average by 60% in all studied brain regions Figure 3. That indicates that the ZF repressed the mutant but not wild-type allele, as expected, under expression with the HSP90 promoter. All transcript levels were normalised to a previously selected (Mielcarek et al., 2013a) panel of brain region specific housekeeping genes Supplementary Figure S3.

**FIGURE 4:**
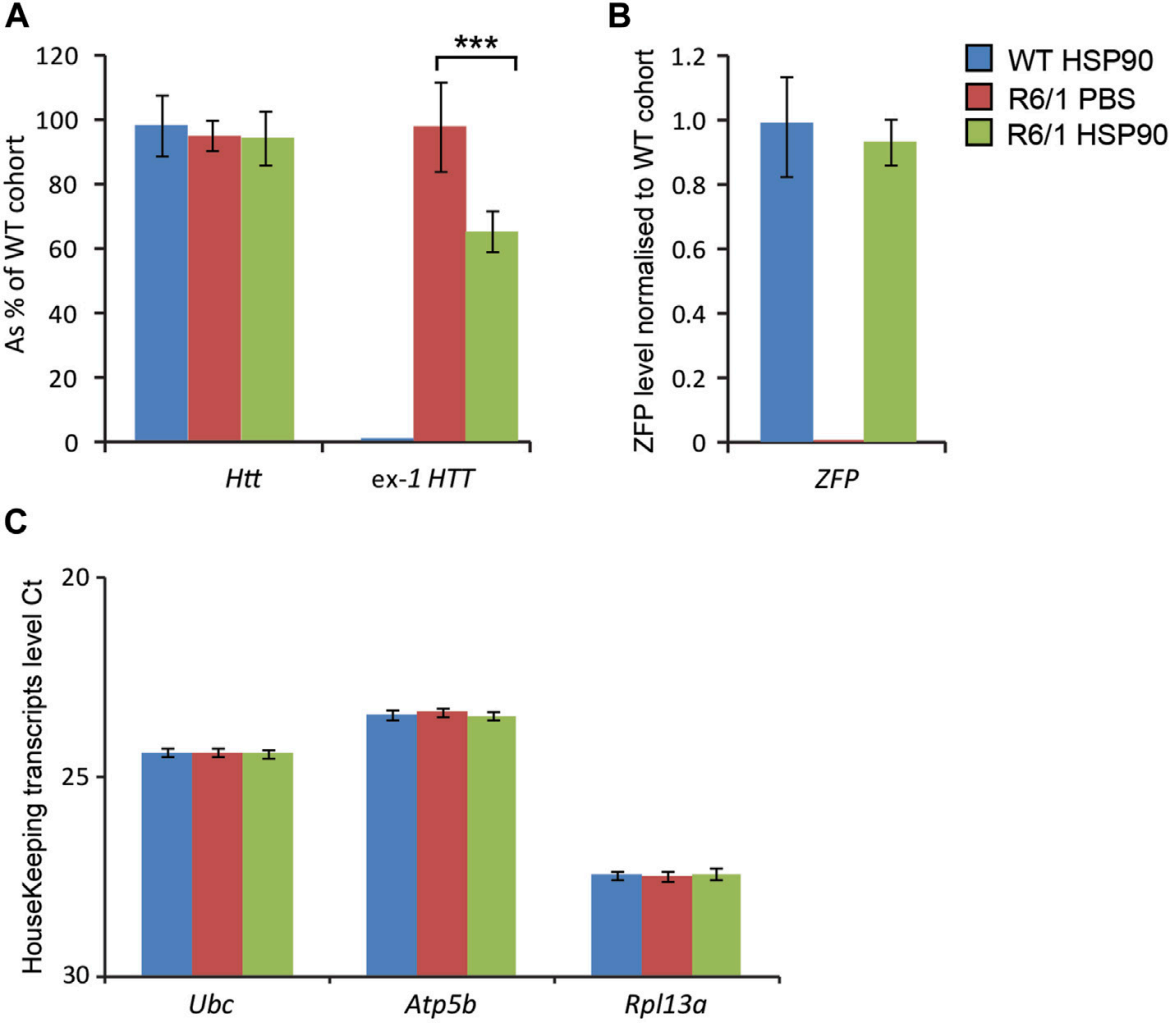
Effects of intrathecal injection of AAV expressing mZF-KRAB under the HSP90 promoter. Three month old wild-type and R6/1 mice were treated either with PBS or with AAV2/9 carrying mZF-KRAB under the HSP90 promoter. **(A)** Unchanged expression WT HTT transcripts as well the repression of mutant Huntingtin mut-Exon1 by the zinc finger (ZFP) expression. mZF-KRAB transcript levels are from whole brains of the WT and R6/1 mice. **(B)** mZF-KRAB transcript levels in the whole brain of wild-type and R6/1 mice. All transcript levels were normalized to three housekeeping genes: **(C)** Raw crossing threshold (Ct) data for a panel of housekeeping genes. The following gene transcripts were used: *Ubc* (Ubiquitin C, 22190), *Atp5b* (ATP synthase subunit, 11947) and *Rpl13a* (Ribosomal protein L13a, 22121). Error bars are ±SEM (*n* = 6). **p < 0.01, ***p < 0.001.

We next studied whether the HSP90 promoter can efficiently drive expression of the mZF-KRAB mRNA in the CNS of symptomatic (3 months of age) R6/1 mice. For this purpose, we changed delivery route and we injected AAV2/9, carrying mZF-KRAB under the HSP90 promoter, into the lower lumbar part of the spine. We found mZF-KRAB mRNA in the whole brain of wild-type and R6/1 mice 6 weeks post a single intrathecal injection Figure 4B. As a result of mZF-KRAB expression, we detected a significant reduction of mutant *Htt* transcripts (approximately 40%) in whole brains of R6/1 mice Figure 4A, while wild-type *Htt* mRNA remained unchanged in both R6/1 mice and their wild-type littermates. This indicates that the zinc finger maintained allele-selective repression under this new promoter and delivery route. The transcripts were normalised to a specific panel of housekeeping genes Figure 4C. Overall, we concluded that our novel minimal HSP90 promoter efficiently drives expression of the therapeutic mZF-KRAB in the R6/1 mouse model during both early postnatal life, as well in the fully symptomatic mice.

**FIGURE 5:**
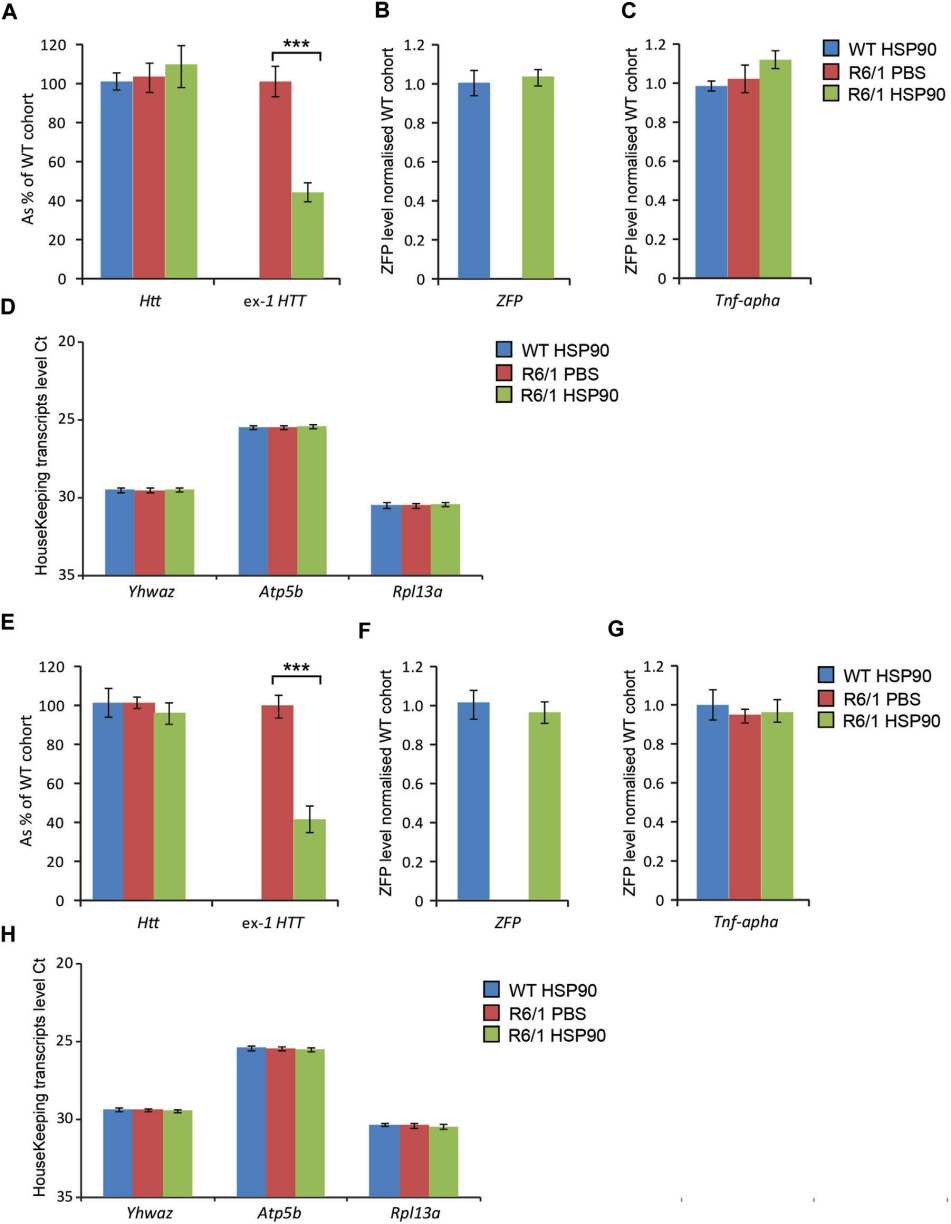
Effects of intramuscular injection of AAV expressing mZF-KRAB under the HSP90 promoter. Tibialis anterior (TA) muscles of the 3 month old wild-type and R6/1 mice were injected with either PBS or with AAV2/9, carrying mZF-KRAB under the HSP90 promoter, and the tissue was harvested either 3 weeks **(A–D)** or 6 weeks **(E–H)** post single injection. The unchanged expression of WT HTT transcripts as well the repression of mutant Huntingtin mut-Exon1 in TA was apparent either 3 weeks **(A)** or 6 weeks **(E)** post single intramuscular injection. There was an apparent expression of the zinc finger mZF-KRAB transcript in the TA muscles of WT and R6/1 mice at both time-points at 3 weeks **(B)** and 6 weeks **(F)**. *Tnf-alpha* (Tumor necrosis factor alpha transcript levels remained unchanged in the TA muscle expressing mZF-KRAB transcripts either 3 weeks **(C)** or 6 weeks **(G)** post single intramuscular injection. All transcript levels were normalized to three housekeeping genes: Raw crossing threshold (Ct) data for a panel of housekeeping genes are presented for the following gene transcripts: *Ywhaz* (Phospholipase A2, 22631), *Atp5b* (ATP synthase subunit, 11947) and *Rpl13a* (Ribosomal protein L13a, 22121). Error bars are ±SEM (*n* = 12). **p < 0.01, ***p < 0.001.

Next, we validated the expression of the mZF-KRAB mRNA driven by HSP90 promoter in HD skeletal muscles. For this purpose, we injected AAV2/9 carrying mZF-KRAB under the HSP90 control directly into tibialis anterior (TA) muscles of 3-month old R6/1 mice (early-symptomatic stage) and their wild-type littermates. In order to verify the mZF-KRAB mRNA expression, we used two cohorts of mice and analysed those tissues at 3 weeks Figures 5A–D and at 6 weeks Figures 5E–H post single injection. We found mZF-KRAB transcripts to be expressed at both time-points (three and 6 weeks post single injection) Figures 5B,F. Already at 3 weeks post injection, the mutant *Htt* transcripts were significantly reduced by 60% as a consequence of the mZF-KRAB expression Figure 5A. Similar levels of mutant *Htt* transcript reduction were detected at 6 weeks post single injection Figure 5E. There was no reduction of wild-type *Htt* mRNA at both time-points, indicating allele-selective repression Figures 5A,E. We also monitored for any potential inflammatory response to the mZF-KRAB in TA muscles by quantification of *Tnf-alpha* (Tumor Necrosis Factor alpha) transcript levels. We did not detect any increase in the *Tnf-alpha* mRNA levels in TA muscles expressing mZF-KRAB at both time points Figures 5C,G. All transcript levels were normalised to a set of previously identified housekeeping genes Figures 5D,H. Our data indicate that the HSP90 promoter efficiently drives expression of the mZF-KRAB therapeutic molecule in the TA muscles of both wild-type and R6/1 mice.

**FIGURE 6:**
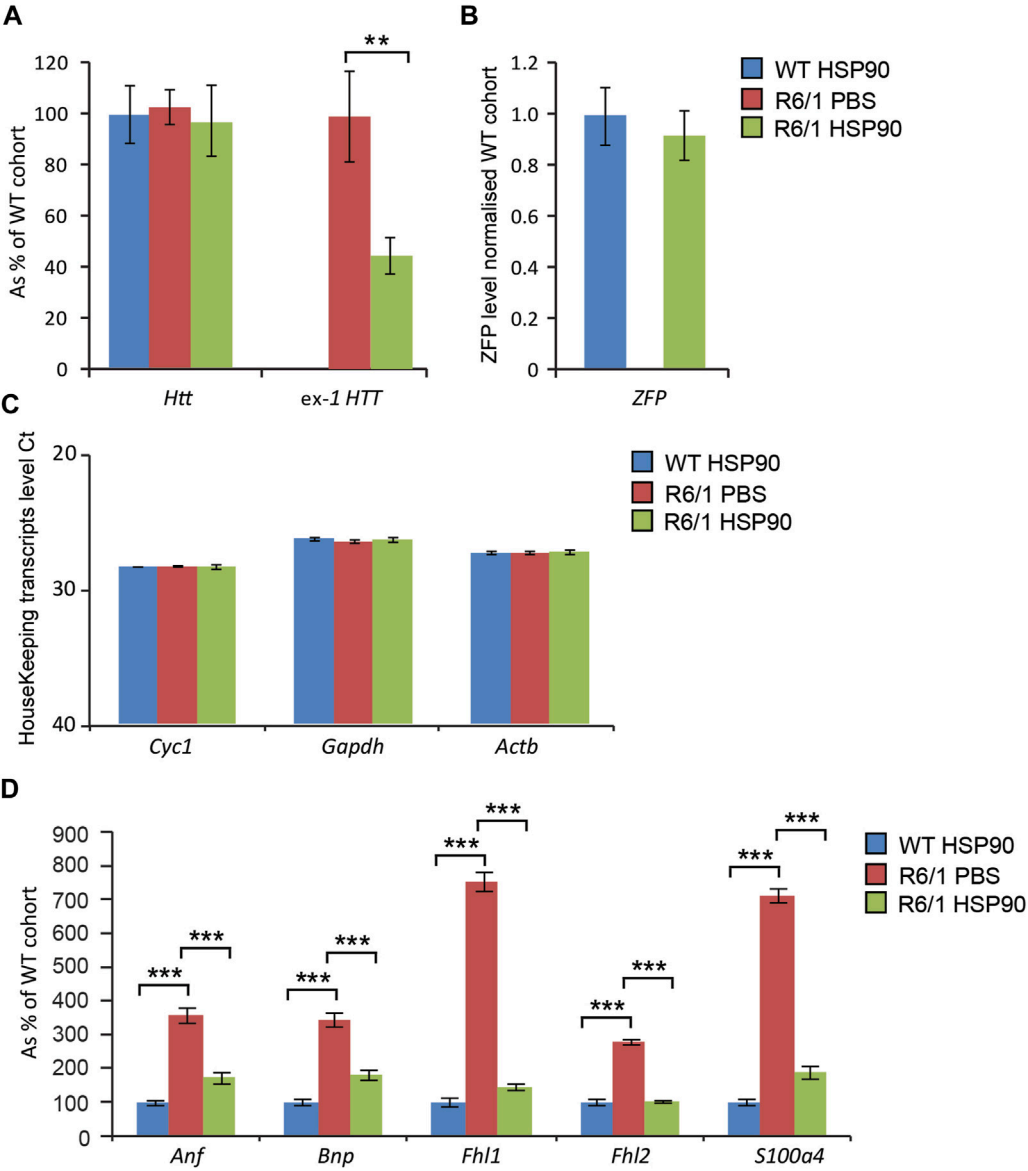
Effects of intrajugular vein injection of AAV expressing mZF-KRAB under HSP90 promoter in the HD heart. Transcript levels of wild type *Htt* remained unchanged while mutant exon-1 *Htt* transcript levels were significantly reduced with zinc finger in the hearts of R6/1mice, in comparison to R6/1 injected with PBS or wild-type mice injected with mZF-KRAB only **(A)**. There was an apparent expression of the zinc finger mZF-KRAB transcript in the hearts of both WT and R6/1 mice at 6 weeks post single injection **(B)**. All transcript levels were normalized to three housekeeping genes **(C)**: Raw crossing threshold (Ct) data for a panel of housekeeping genes are presented for the following gene transcripts: *Cyc1* (Cytochrome c-1, 66445), *Atcb* (Actin, beta, cytoplasmic, 11461), *Gapdh* (Glyceraldehydes-3-phosphate dehydrogenase, 14433) **(D)**. A number of biomarkers of the HD pathology in the heart have been significantly reversed: *Anf* (atrial natriuretic factor), *Bnp* (brain natriuretic protein), members of the four-and-a-half LIM family *Fhl1* and *Fhl2, S100A4S100* calcium binding protein A4). Error bars are ±SEM (*n* = 6). **p < 0.01, ***p < 0.001.

HD-related cardiomyopathy has been described as a relatively late pathological event in HD mouse models (Mielcarek et al., 2014a; Zielonka et al., 2014b). Hence, we validated the activity of the therapeutic mZF-KRAB, driven by the HSP90 promoter in symptomatic R6/1 mice, at 6 months of age. Heart tissue was collected 6 weeks post a single intravenous (jugular vein) injection with AAV2/9 carrying the mZF-KRAB under HSP90 promoter control. The mZF-KRAB expression was clearly detectable in hearts of R6/1 mice and their wild-type littermates Figure 6B. Similarly to the CNS and skeletal muscles, mZF-KRAB significantly reduced mutant *Htt* mRNA levels by 60%, while wild-type *Htt* transcripts remained unchanged Figure 6A. Since, there are no previous reports regarding any therapeutic effects of silencing mutant *Htt* in HD hearts, we performed a quantitative analysis of previously established biomarkers linked to the HD related cardiomyopathy in HD mouse models (Mielcarek et al., 2014a). We found that *Anf* (atrial natriuretic factor) transcripts as well *Bnp* (brain natriuretic protein) mRNAs were significantly downregulated to the level detected in the wild-type mice Figure 6D. Similarly, the expression level of two members of the four-and-a-half LIM family *Fhl1* and *Fhl2* were significantly reversed to the level observed in wild-type mice Figure 6D. Finally, transcripts of *S100A4* (S100 calcium binding protein A4) gene were reduced by 5-fold in comparison to R6/1 mice injected with PBS as a control, although the *S100A4* mRNA level was still significantly higher than in wild-type mice Figure 6D. All transcripts were normalised to a previously established panel of housekeeping genes (Mielcarek et al., 2014a) Figure 6C. Thus, our study shows that the HSP90 promoter can also efficiently drive expression of mZF-KRAB therapeutics in the heart tissue of HD mice.

## Discussion

Gene locus silencing technology is based on the activity of synthetic zinc finger (ZF) molecules that can act as selective repressors to target virtually any gene sequence, resulting in a broad therapeutic potential. Due to their relative small size, these active ZF molecules can be delivered to various tissues and cells with adeno associated viruses (AAV) and their expression can be regulated by either tropism of the AAV or a cell-selective promoter (Au et al., 2021). In the past, we have shown that these synthetic ZF molecules can efficiently and very specifically target the mutated form of the HTT gene, to significantly lower its expression in the CNS of various HD mouse models, in an allele-selective manner (Garriga-Canut et al., 2012; Agustin-Pavon et al., 2016).

Allele-selectivity by HTT-repressing ZFs is based on the properties of zinc fingers that bind longer target sequences preferentially because of avidity and co-operativity effects. For illustration, distributions of mutant Huntingtin in human populations have modal values of ∼15 CAG repeats for wild-type alleles and ∼42 for the longer mutant alleles. Thus, the mutant target provides many more overlapping opportunities for a ZFP to bind, increasing the avidity of the interaction. Moreover, it is well-known that zinc fingers unwind the DNA helix slightly when they bind (Shi and Berg, 1996), facilitating binding reactions by subsequent zinc fingers, both within a zinc finger chain and for multiple chains binding to a longer target. The DNA unwinding is essential for the proper alignment of the DNA-contacting amino acid residues and the interaction sites on DNA. These effects lead to co-operativity in zinc finger binding, leading to higher apparent affinity for longer mutant DNA-repeat targets.

*In vivo*, specifically targetting mutant HTT in the CNS led to an amelioration of a number of molecular and neurological phenotypes in HD mouse models and became a valid therapeutic strategy for Huntington’s disease (Garriga-Canut et al., 2012). However, the therapeutic effect of ZF molecules was limited to relatively short period of time, likely due to a methylation of the synthetic CAG promoter (Zhou et al., 2014) that was used to drive expression of ZF *in vivo* (Garriga-Canut et al., 2012). In fact by switching from the CAG promoter to an endogenous Neuronal Specific Enolase promoter (NSE), we very significantly improved expression of therapeutic ZF molecules over time in the CNS of HD mouse models and were able to observe the therapeutic effect up to 6 months post single injection (Agustin-Pavon et al., 2016). Although HD is primarily recognised as a neurological disorder, the last decade of extensive studies discovered a number of pathological events occurring in the peripheral organs especially in skeletal muscles (Zielonka et al., 2014a; Mielcarek and Isalan, 2015) and heart (Zielonka et al., 2014b; Critchley et al., 2018). In fact, myostatin inhibition in HD skeletal muscles was by itself sufficient to improve a number of molecular and physiological features in the presence of ongoing CNS degeneration in an HD mouse model (Mielcarek et al., 2014b). Hence, HD has been confirmed as a multi-system disorder (Mielcarek, 2015; Mielcarek and Isalan, 2021a) and it is becoming apparent that an efficient gene therapy for HD should also be design to target mutant HTT very widely in a number of peripheral tissues and organs. Thus, in this study, we aimed to identify a novel minimal endogenous promoter-enhancer that can efficiently drive expression of therapeutic ZF molecules in various somatic HD tissues.

One of the prominent pathological features in HD is transcriptional deregulation that has been described as an early and progressive event (Hervas-Corpion et al., 2018). These transcriptional changes have been characterised by a general downregulation of a number of gene sets, likely by altering levels of multiple gene expression regulators (Valor, 2015) or by unbalanced epigenetic regulation (Francelle et al., 2017). Hence, in order to identify a ubiquitous promoter that can be active during lifetime of HD, we screened a number of transcription profiling datasets to identify stably-expressed transcripts from early to end-stage of the disease. This approach led us to identify a minimal HSP90 promoter which we then validated for its ability to drive expression of a therapeutic ZF in various somatic tissues, at both pre-symptomatic and symptomatic stages in an HD mouse model. Firstly, we found that ZF expression, controlled by our novel HSP90 promoter, can last at least 6 months in the whole brain, after a single injection into neonatal R6/1 mice, in a similar manner to the previously characterised NSE promoter (Agustin-Pavon et al., 2016).

Next, we used the more clinically-relevant intrathecal route to deliver ZF molecules, under the control of HSP90 promoter, into the CNS of symptomatic R6/1 mice. We found that 6 weeks after a single injection, there was a significant reduction of mutant HTT mRNAs in the whole brain, indicative of active ZF therapeutics being present. Hence, we concluded that this new HSP90 promoter can efficiently drive expression of ZF molecules even with intrathecal delivery into symptomatic R6/1 mice.

Since malfunction of skeletal muscles is a major pathological feature of HD (Mielcarek et al., 2015), we further assessed the ability of HSP90 to drive ZF expression in the tibialis anterior, in symptomatic R6/1 mice. We successfully detected ZF transcripts with a similar beneficial effect as in the CNS: approximately 60% reduction in mutant HTT transcript levels. This is the first study to show an efficient reduction in mutant HTT mRNA levels in HD skeletal muscles *in vivo*. Importantly, the expression of ZF molecules in HD skeletal muscles did not trigger any apparent immunological response, as judged based on unchanged *Tnf-alpha* transcript levels. Finally, we addressed the feasibility of HSP90 promoter use in cardiac tissue. We analyzed heart tissues from symptomatic R6/1 mice at 3 and 6 weeks post single injection of AAV2/9, carrying ZF molecules under the HSP90 promoter control. We found ZF transcripts to be abundantly expressed in the HD hearts, leading consequently to a significant reduction of mutant *Htt*. We also verified whether mutant Htt reduction might have an impact on the previously characterised panel of biomarkers related to HD induced cardiomyopathy (Mielcarek et al., 2014c). Strikingly, we found that transcript levels of *Anf* (atrial natriuretic factor), *Bnp* (brain natriuretic protein), two members of the four and half LIM family *Fhl1* and *Fhl2* and *S100A4* (S100 calcium binding protein A4) were brought back nearly to wild-type levels. This indicates that lowering mutant HTT transcript levels directly in HD hearts leads to reversing transcriptional pathological remodelling. However, there is a need in the future to further validate whether other pathological features of HD cardiomyopathy can be also efficiently reversed (Mielcarek et al., 2014c; Toczek et al., 2016a; Toczek et al., 2016b).

In summary, our study offers a novel asset, an HSP90 (HSP90AB1) promoter-enhancer, to be used for the efficient expression of therapeutic molecules in various somatic tissues. The use of this promoter can be extended beyond the HD therapeutic area whenever there is a need to apply therapeutic molecules in a ubiquitous manner.

## Materials and methods

### Mouse maintenance and genotyping

The R6/1 mouse line was purchased from Jackson Laboratories (US) and was bred and genotyped as previously described (Mielcarek et al., 2013b; Agustin-Pavon et al., 2016; Mazur-Michalek et al., 2022). The cumulative CAG count for all R6/1 mice used in this study was 131 ± 2.7 SD. All experimental procedures were conducted under a project license from the Home Office, UK and approved by the Animal Welfare and Ethical Review Body of Imperial College London. All animals had unlimited access to water and breeding chow (Special Diet Services, Witham, UK), and housing conditions and environmental enrichment were as described previously (Agustin-Pavon et al., 2016).

### AAV production

AAV2/9 mZF–KRAB, containing a HSP90 promoter were used in this study were produced at the Centre for Animal Biotechnology and Gene Therapy of the Universitat Autonoma of Barcelona, as described previously (Agustin-Pavon et al., 2016). The AAV was purified by precipitation with PEG 8000, followed by iodixanol gradient ultracentrifugation with final titers up to ∼10^12^ genome copies/mL.

### Mouse surgery—AAV delivery routes

Free hand intraventricular AAV injections in neonates were performed as previously described (Kim et al., 2014; Agustin-Pavon et al., 2016). Briefly, neonatal mice (P0.5) were anesthetized with isoflurane and were subjected to bilateral intraventricular injection of AAVs, within 24 h of birth to ensure full ventricular dilation. The maximum possible volume of 2 μl of viral vector, or PBS, was injected into each cerebral lateral ventricle, using a sterile 10 μl Hamilton microsyringe. The injection site was at the 2/5 of the distance from the lambda suture to each eye and the needle was inserted at a depth of approximately 3 mm. Warming pads were used to recover neonatal mice immediately after injection, as described previously (Agustin-Pavon et al., 2016). In this study we injected 4 μl in total per mouse with a viral titre of 10^10^ AAV/μl (4 × 10^10^ viral particles total). Mice were killed at 3, 6, 12 or 24 weeks after the injections and brains were harvested, snap frozen in liquid nitrogen, and stored at −80°C until further analysis by qRT-PCR.

The protocol for Intrathecal injections (IT) was adopted from (Vulchanova et al., 2010; Njoo et al., 2014). Adult mice were anaesthetized by inhalation of a mixture of 1.0 l/min O_2_ and up to 5.0% isoflurane. Anaesthesia was confirmed by lack of movement after squeezing a paw and slow-down respiration. AAVs or PBS were injected into the intrathecal space of the lower lumbar cord. The successful 27 G needle penetration into the intrathecal space was indicated by a tail flick. The maximum volume of the injected solution was 10 μl per mice. In total 10^10^ viral particles was injected per mouse. Next, mice were recovered immediately after injection and placed on the warming pads. Mice were killed 6 weeks after the injections and brains were harvested, snap frozen in liquid nitrogen, and stored at −80°C until further analysis by qRT-PCR.

Direct intra-muscular injections (IM) were performed accordingly to the previously published protocol (Gruntman et al., 2013). Prior to surgery, mice were weighed and deeply anaesthetized by inhalation of a mixture of 1.0 l/min O_2_ and 5.0% isoflurane. Anaesthetized mice were taken out of anaesthesia chamber and the anaesthetic mixture was lowered to 0.5 l/min oxygen and 1.0%–2.0% isoflurane and provided through the flow mask. Typically, tibialis anterior muscles were directly injected with a 29-Gauge 0.5 ml insulin syringe (BD). A maximum of 10 μl of AAVs or PBS was injected. In this study we injected total 4 × 10^10^ viral particles per muscle. Mice were killed at 3 and 6 weeks after the injections and TA muscles were harvested, snap frozen in liquid nitrogen, and stored at −80°C until further analysis by qRT-PCR.

In order to express the zinc finger transcripts in the HD heart, intra-jugular injections (IJ) were performed according to the previously published protocol (Gao et al., 2020). Briefly, mice were weighed and deeply anaesthetized by inhalation of a mixture of 1.0 l/min O_2_ and 5.0% isoflurane. Anaesthetized mice were placed in a ventral recumbent position and the anaesthetic mixture was lowered to 0.5 l/min oxygen and 1.0%–2.0% isoflurane. A small incision was made lateral to the ventral midline, from the pectoral muscle to the lower neck. The right jugular vein was exposed with blunt dissection. AAV vectors or PBS were delivered into the systemic circulation through a direct injection using a 29-Gauge 0.5 ml insulin syringe (BD) into the right jugular vein. In this study we injected a total of 10^11^ viral particles per mouse. Mice were killed at 6 weeks after the injections and hearts were harvested, snap frozen in liquid nitrogen, and stored at −80°C until further analysis by qRT-PCR.

A summary of all delivery routes including time-lines can be found in the Supplementary Figure S4.

### RNA extraction and taqman real-time PCR expression analysis

Total RNA from eye tissues was extracted with the mini-RNA kit (Qiagen, United Kingdom), according to the manufacturer’s instructions. The reverse transcription reaction was performed using MMLV superscript reverse transcriptase (Invitrogen, United States) and random hexamers (Sigma, United States), as described in earlier studies (Agustin-Pavon et al., 2016; Piotrowska et al., 2017; Mazur-Michalek et al., 2022). All Taqman qPCR reactions were performed with a LightCycler^®^ 480 Instrument (Roche), as described previously (Mielcarek and Isalan, 2021b; Mazur-Michalek et al., 2022). Estimation of mRNA copy number was determined in triplicate for each RNA sample by comparison with the geometric mean of three endogenous housekeeping genes (Primer Design, United Kingdom), as described previously for the brain and brain regions (Mielcarek et al., 2011; Mielcarek et al., 2013b; Agustin-Pavon et al., 2016), skeletal muscles (Mielcarek et al., 2015; Mielcarek et al., 2017) and heart tissue (Mielcarek et al., 2014a; Mielcarek et al., 2014c; Toczek et al., 2016b). The following Taq-man assays for selected genes of interest, were used as previously described: mZF, wt HTT and mutant exon-1 HTT (Agustin-Pavon et al., 2016), HD heart biomarkers (Mielcarek et al., 2014c).

### Statistical analysis

Values were presented as mean ± SEM. Statistical analysis was performed using paired Student *t* tests (Excel) or One-Way Anova SPSS (IBM). A *p*-value of 0.05 was considered as a significant difference.

## Data Availability

The original contributions presented in the study are included in the article/Supplementary Material, further inquiries can be directed to the corresponding author.
