# Supplementary material for: A minimal region of the HSP90AB1 promoter is suitable for ubiquitous expression in different somatic tissues with applicability for gene therapy
Source: Front Mol Biosci. 2023 Apr 17;10:1175407. doi: 10.3389/fmolb.2023.1175407 (PMC10149993; doi:10.3389/fmolb.2023.1175407)
Supplement: Supplementary file 1 [file DataSheet1.PDF]

Supplementary Materials

A minimal region of the HSP90AB1 promoter is suitable for ubiquitous expression in different somatic tissues with applicability for gene therapy.

Michal Mielcarek and Mark Isalan

This file includes:

Supplementary Figures 1-4

Key

gctagc= NheI cloning sites (added)
gatagc= cryptic NheI site (deleted)
Transcription start site (TSS) in **BOLD CAPITALS.**
ctcgagaccATGG = leader sequence and Kozak-context start codon (ATG)

>hsp90ab1_promoter
gctagcaacaccctagggccttctgagcaatcctacccagtgtctcctcatatattgatttctttatgggcttcacacacacacacacacacac
acacacacacacacacacacacacacagaattaaggagaggctaacagacagtgcaggatgggatgataacagacgaagtagacagaggcaagg
agaaagcaactactgtttaacaatgaatgcacattagacagactgcaggcaagcaccgggaacaaaggtgtgggcggtggtgtggggacacaag
ccagcatgagctaagatagcagagcactgagtgcccatcctctactggagggctcatcagtccaacaagcttccagatgcagccttggaaaaag
gcaaggctagattgccagctgaaggacatggcaggccacctttagaacagaggcactggcacaacttggttttctggctcctggaactgggcca
acctgtgaccagcaccttcatgcggatgcctagaactccagcttctctgaaaagactgggacctgctcctctctaggtccaaagagctgcatgc
agtagggaagaggctagagaagcgaaaccagcttgagaaacagcttgtgctcacatagggagggcgcacgtacccgcgcgctgtgtacgtggga
gaccggggaggctgaggggtggggagtgttctacccagtagcgcaagctgatagctcggttctctgttcactagaaggtgtccgcagtcactca
cccccacagcccccgtgccctgtgaccgatccaggtcagctatccctccctctgcgctccactcccccactgttatgtgggcctcttagggcca
cgcgtggagggtcgttcaaccctggcccacggtaggcagacttggggaaaatttcttcccagggtaagatcaaggtaggggaaaaaaaaaaaa
aaaaaagccacccagccaagcggcgacgaagacactgcccccgccgcagcaggggaggtggagcctagggggagggtggagaccgccgagac
aggcctagaaactgctggaagaaatcgcagcaccaccgctgctgatccttccgccgcaggccgccaaagagtccctaccagcccaggcccgtgc
ccctcccctcggggaaagcggctcccagcctgaagctgtgctgtacccgggagggtggggatgggggaatcgggggcctccttaaagttggaca
aggaatttatcatcctttttctcttgatgtgcgatttgtagggaacattctagtaagatcgggtctggaaatggcagccgagttggccacctcca
ttctctttcagtcccctgagttctggactcttggggggtgggggggtggaagcgcctaccttgagttttctgaggcagtccgtagggtattcgc
ccgcagatacatccctaattgcatatgcatgctccctgctcatcttgagggggacatgtcctactcctgcagaaatgggggatgtgcaaaacg
atattgaattggccttgactcaggaaccaggcccggggtcccgctcctccccgcccccctccacgatctgctaccatgacgtcaaggtgggcggg
cggcggcaggtgcgtggcccgcagccactcctttaaggcggagggatccaagggcggggcccgggctgtgcttcgccttatatagggcggtcgg
gggcgttcgggagct**CTCTTGAGTCACCCCCGCGCAGCCTAGGCTTGCCGTGCGAGTCGGACTTGGTCCGGGCCCACCACCCTGCTCTGTACTA**
CTACTCGGCTTTCCCGTCAAGgctagcctcgagaccATGG

Supplementary Fig S1. Sequence of the HSP90AB1 promoter-enhancer used in this study. The sequence is shown together with a Key, highlighting important features of the construct. For transgene expression, a Kozak-context start codon is placed downstream of the last NheI site.

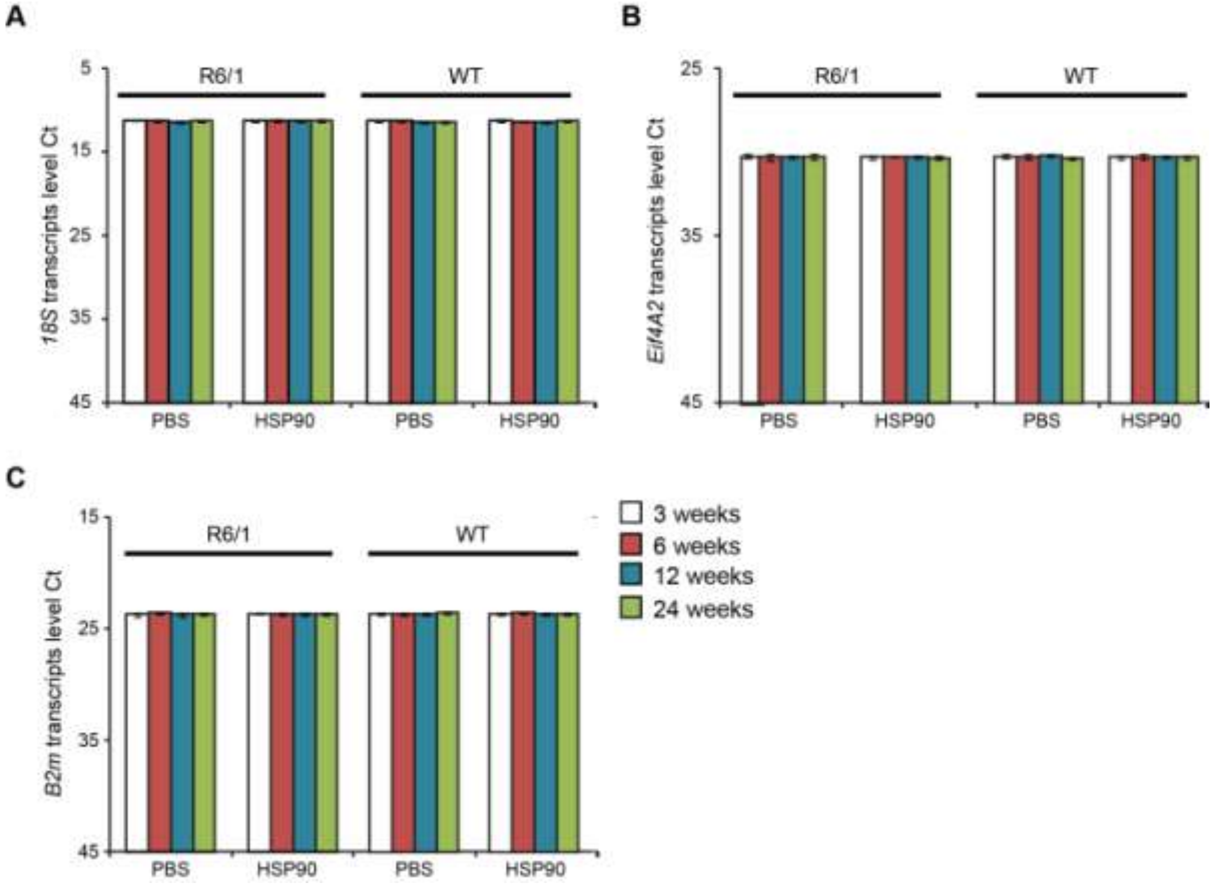

Supplementary Figure S2. Reference genes used for qPCR from whole brain of the R6/1 and WT mice spanning all exercised time points at 3, 6, 12 and 24 weeks. Raw crossing threshold (Ct) data for a panel of housekeeping genes in WT and R6/1 mice are presented from **(A)** *18S* (18S rRNA, 19791) **(B)** *Eif4A2* (Eukaryotic translation initiation factor 4A2, 13682) **(C)** *B2m*, (Beta-2-microglobulin, 12010). Error bars are ± SEM (n = 4).

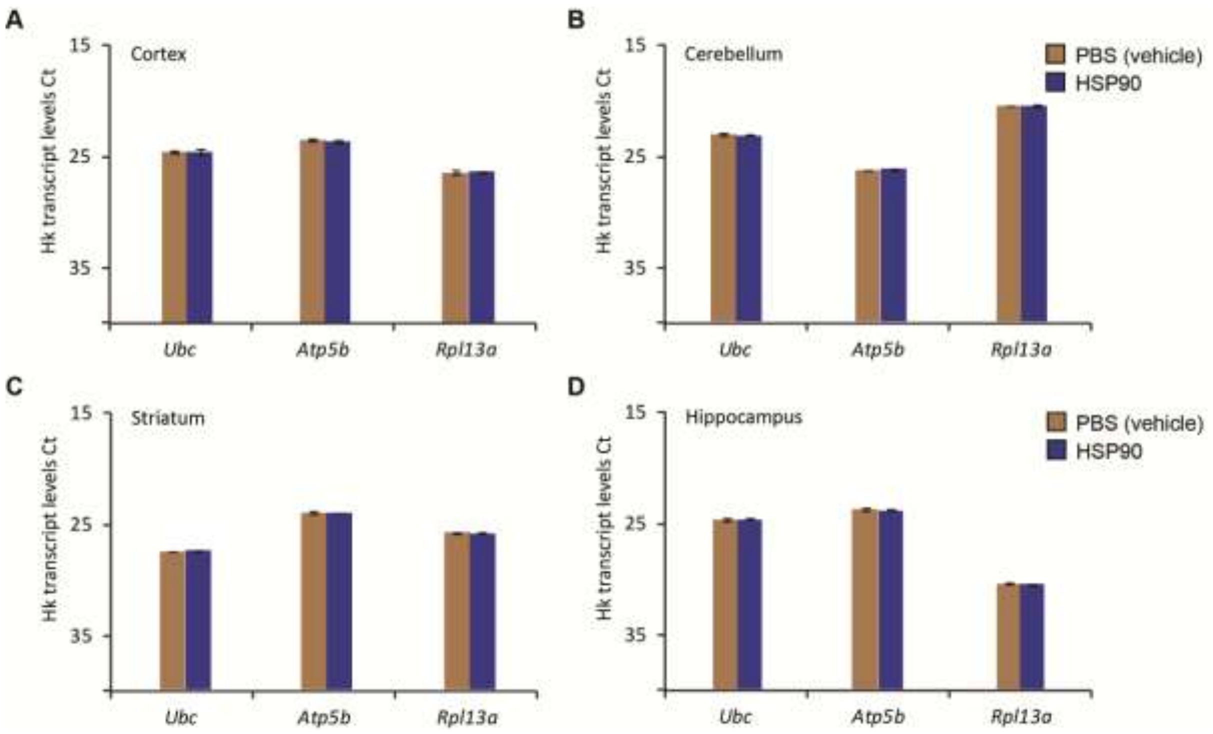

Supplementary Figure S3. Reference genes used for qPCR from specific brain regions of the R6/1 mice. Raw crossing threshold (Ct) data for a panel of housekeeping genes in R6/1 mice. The following gene transcripts were used: *Ubc* (Ubiquitin C, 22190), *Atp5b* (ATP synthase subunit, 11947) and *Rpl13a* (Ribosomal protein L13a, 22121) in specific brain regions: **A)** cortex, **B)** cerebellum, **C)** striatum, **D)** hippocampus. Error bars are ± SEM (n = 4).

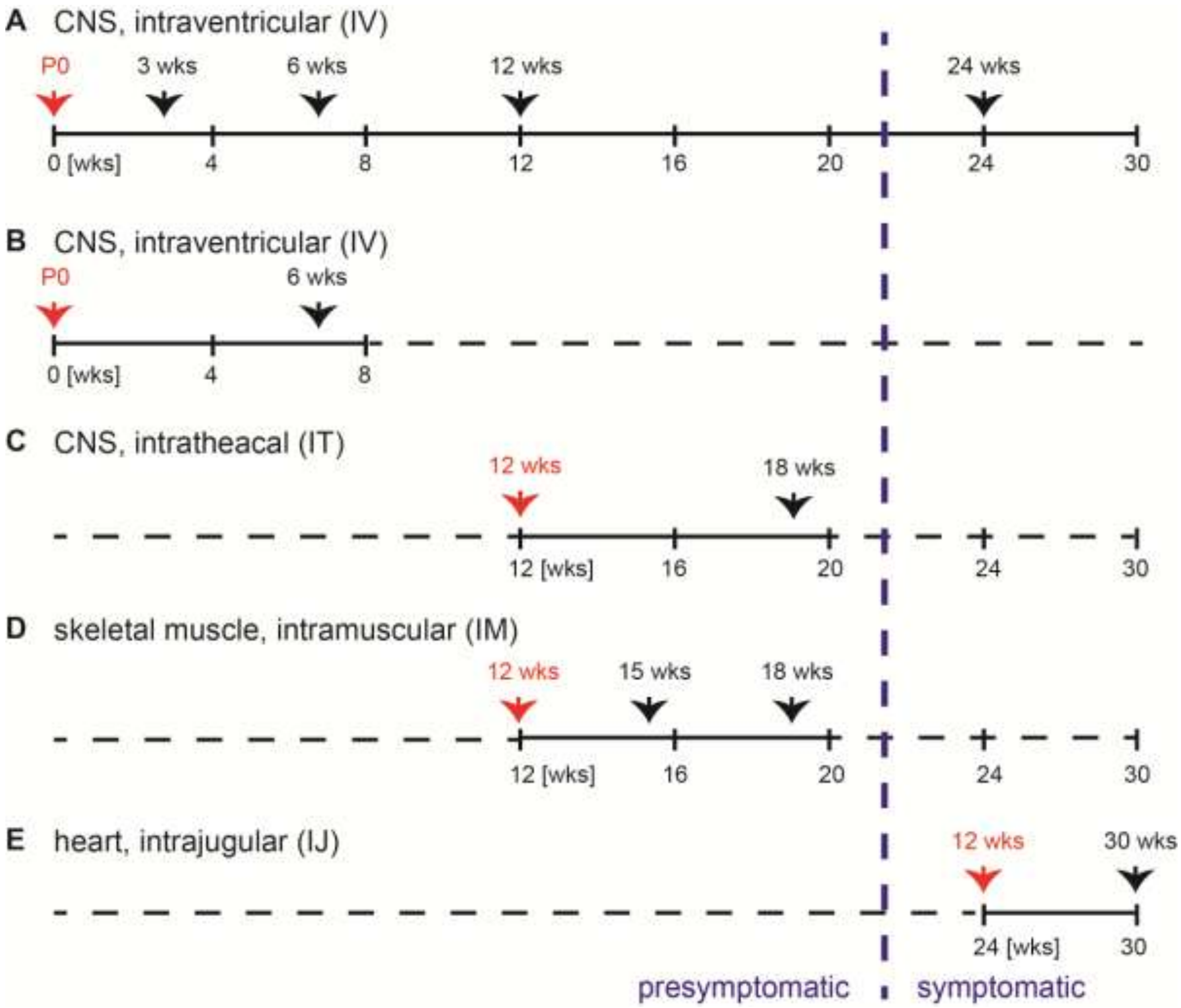

Supplementary Figure S4. A summary of the experimental design for this study. **(A)** a single intraventicular injection (IV) at P0 refers to results presented in Figure 1; **(B)** a single intraventricular injection at P0 refers to results presented in Figure 2; **(C)** a single intrathecal (IT) injection at 12 weeks (3 months) of age refers to results presented in Figure 3; **(D)** a single intramuscular injection (IM) re refers to results presented in Figure 4; **(E)** a single intrajugular injection (IJ) to results presented in Figure 5. Presymptomatic stage (0 -20wks), symptomatic stage (20 wks >), weeks (wks).
